# A Study of Diagnostic Accuracy Using a Chemical Sensor Array and a Machine Learning Technique to Detect Lung Cancer

**DOI:** 10.3390/s18092845

**Published:** 2018-08-28

**Authors:** Chi-Hsiang Huang, Chian Zeng, Yi-Chia Wang, Hsin-Yi Peng, Chia-Sheng Lin, Che-Jui Chang, Hsiao-Yu Yang

**Affiliations:** 1 Department of Anesthesiology, National Taiwan University College of Medicine, Taipei 10051, Taiwan; chhuang@ntu.edu.tw (C.-H.H.); yichiawang@ntu.edu.tw (Y.-C.W.); 2 Department of Anesthesiology, National Taiwan University Hospital, Taipei 10048, Taiwan; 3 Institute of Occupational Medicine and Industrial Hygiene, National Taiwan University College of Public Health, Taipei 10055, Taiwan; michellzeng0912@gmail.com (C.Z.); yope159@gmail.com (H.-Y.P.); L1204366@gmail.com (C.-J.C.); 4 Department of Clinical Laboratory Sciences and Medical Biotechnology, National Taiwan University, Taipei 10051, Taiwan; b04404046@ntu.edu.tw; 5 Department of Family Medicine, National Taiwan University Hospital, Taipei 10048, Taiwan; 6 Department of Public Health, National Taiwan University College of Public Health, Taipei 10055, Taiwan

**Keywords:** sensor array, lung cancer, electronic nose

## Abstract

Lung cancer is the leading cause of cancer death around the world, and lung cancer screening remains challenging. This study aimed to develop a breath test for the detection of lung cancer using a chemical sensor array and a machine learning technique. We conducted a prospective study to enroll lung cancer cases and non-tumour controls between 2016 and 2018 and analysed alveolar air samples using carbon nanotube sensor arrays. A total of 117 cases and 199 controls were enrolled in the study of which 72 subjects were excluded due to having cancer at another site, benign lung tumours, metastatic lung cancer, carcinoma in situ, minimally invasive adenocarcinoma, received chemotherapy or other diseases. Subjects enrolled in 2016 and 2017 were used for the model derivation and internal validation. The model was externally validated in subjects recruited in 2018. The diagnostic accuracy was assessed using the pathological reports as the reference standard. In the external validation, the areas under the receiver operating characteristic curve (AUCs) were 0.91 (95% CI = 0.79–1.00) by linear discriminant analysis and 0.90 (95% CI = 0.80–0.99) by the supportive vector machine technique. The combination of the sensor array technique and machine learning can detect lung cancer with high accuracy.

## 1. Introduction

Lung cancer is the leading cause of death worldwide, accounting for 1.69 million deaths in 2015 [1]. Chest radiography (CXR) and low-dose computed tomography (LDCT) are commonly used in the screening of lung cancer. However, screening with CXR does not reduce lung cancer mortality [2] due to the high number of false-negative results [3]. Aside from high dose radiation exposure, LDCT also has problems with the high number of false-positive results that induces subsequent psychological stress. The U.S. Preventive Services Task Force recommends that LDCT is only suitable for selected elderly subjects (aged 50 years or older) with a heavy cigarette smoking history (20 pack-years or more) [4]. The development of a screening method for the general public is therefore warranted.

Oxidative stress plays an important role in the pathogenesis of lung cancer, as it increases the generation of reactive oxygen species and the lipid peroxidation of polyunsaturated fatty acids [5] and produces specific volatile organic compounds (VOCs) [6]. Specific VOCs, such as ethanol and isopropanol [7,8], may be released and analysed as breath tests for the detection of lung cancer. Common breath tests for lung cancer include an electronic nose (E-nose) and gas chromatography-mass spectrometry (GC-MS) [9,10]. The E-nose has the advantages of a short analysis time, low cost and easy operability compared to GC-MS [11]. Among the different types of sensors, chemical sensors can detect VOCs at the parts per billion (ppb) level [12]. The objective of this study was to develop a breath test for the detection of lung cancer using a chemical sensor array and machine learning technique.

## 2. Materials and Methods

We conducted a prospective study to plan sample collection and followed the STARD guideline to report the diagnostic accuracy in this study [13].

### 2.1. Participants

We conducted a case-control study performed from July 2016 to May 2018, and recruited study subjects from National Taiwan University Hospital. The cases were lung cancer patients who received surgery and confirmed by a pathological report. Using density sampling, we recruited controls from subjects who had no history of cancer and received surgery for gall bladder stones, hernia, haemorrhoid resection, anal fistula and varicose vein surgery, appendectomy, or tympanoplasty in the same hospital during the same period. The lung cancer staging was in accordance with the American Joint Committee on Cancer’s TNM classification (7th edition) [14]. Frozen tissues of the surgical specimens and were examined for histology with haematoxylin and eosin staining [15]. The protocol in this study was approved by the Research Ethics Committee of National Taiwan University Hospital (No. 201512160RINC), and all subjects provided informed consent before enrolment.

### 2.2. Exclusion Criteria

Pregnant women and people less than 20 years of age were excluded from enrolment in this study. To prevent confounding factors from other diseases or chemotherapy, subjects with benign lung tumours, metastatic cancer, carcinoma at another site, carcinoma in situ, or minimally invasive adenocarcinoma; subjects receiving chemotherapy; and subjects who had chronic obstructive lung disease (COPD), asthma, or diabetes controlled by medication were excluded from the statistical analysis.

### 2.3. Test Methods

#### 2.3.1. Collection of Alveolar Air Breath Samples

**Figure 1:**
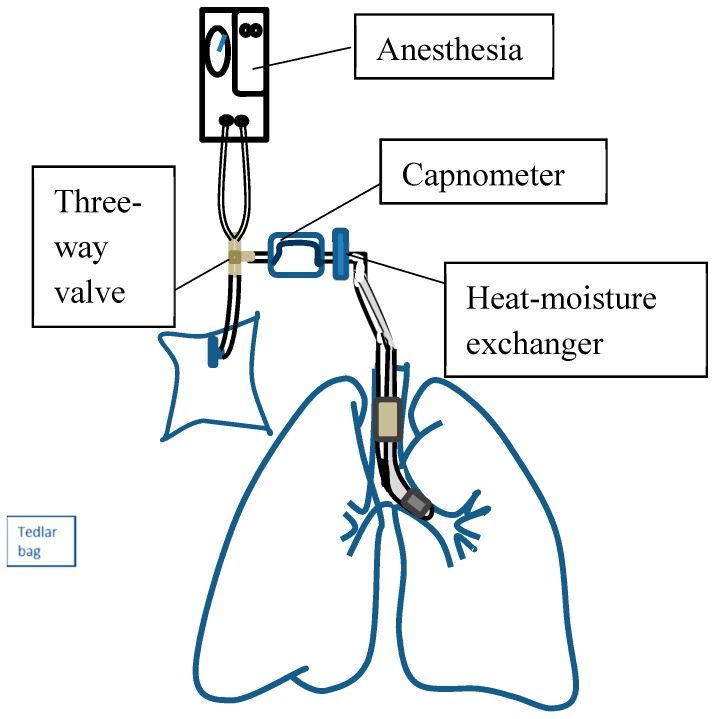
Schematic of the system framework and sample collection.

We collected the alveolar air samples using a standardized procedure [16]. Because VOC concentrations may be affected by diet, flow rate, and anatomical dead space [17,18], all subjects were required to stop eating and smoking for 12 h before the air sampling. The air was then taken after intubation with an endotracheal tube and before surgery. To prevent contamination from the upper airway, we sampled alveolar air from the endotracheal tube with a capnometer (Masimo, Irvine, CA, USA). Under the visual control of a carbon dioxide-controlled sampling device, the alveolar air was taken from the breathing circuit during the alveolar phase of expiration [19] (Figure 1). To maintain a consistent flow rate of 125 mL/s, we set the ventilator to a tidal volume of 500 to 600 mL, a respiratory rate of 8–10/min, and an inspiratory-to-expiratory time ratio (I:E) of 1:2. To decrease the influence of humidity, all breath samples were dehumidified by a heat-moisture exchanger and then collected in a 1-L Tedlar bag (SKC Inc., Eighty Four, PA, USA).

#### 2.3.2. Measurement Set-Up

We collected the alveolar air and analyzed samples within two hours. The experimental setup for the analysis of alveolar air consisted of an E-nose, computer, three-way valve and Tedlar bag (Supplementary Material, Figure S1). The bags were connected with the necessary fixture, including an airtight PVC tube and a three-way valve for connection to the E-nose. According to the *E-nose* instructions, the setting comprised 10 s of a baseline purge and 40 s of the sample purge, which was sufficient for most sensors to reach the steady-state, followed by 10 s of a wash-out to return to the baseline (Supplementary Material, Figure S2). The E-nose flow rate was set to 120 cc/min, and the breath sample in each bag was analysed 10 times. We carefully examined the raw sensor responses and deleted drifted data. Then, we deleted the first measurement and obtained a mean value of the remaining measurements [20]. All procedures were performed in the same room with air conditioning to maintain a constant temperature and humidity.

### 2.4. Sensors

We used the E-nose chemical sensor Cyranose320 (Sensigent, Baldwin Park, CA, USA), composed of 32 nanocomposites conducting polymer (CP) sensors, to analyse the breath samples. CP sensors consist of highly sensitive carbon nanotubes. Readers can find the photos of the sensor published by Lu et al. [21]. The E-nose sensitivity was at the parts per million to billion (ppm-ppb) level. The sensor polymers consist of the poly(vinyl butyral), poly(vinyl acetate), poly(styrene), poly(ethylene oxide). When the sensors are exposed to a mixture of VOCs, the polymers will swell and change the electrical resistance [22]. The room air pumped into the E-nose was analysed to provide the baseline sensor response (*R*_0_). The raw data were normalized and autoscaled to eliminate background noise and exclude outliers [21,23] and then used to derivate the prediction model:

(1) $$\mathrm{Sensor}\;\mathrm{response}:\frac{\Delta R}{R_{0}}=\frac{\left(R_{max}-R_{0}\right)}{R_{0}}$$

The raw data were normalized using the following equation:

(2) $$\sum_{k=1}^{\mathrm{NV}}x_{ik}^{2}=c_{i}$$

where *k* designates the sensor, *i* designates the samples of study subjects, and NV is the total number of sensors. Then, the data were autoscaled to the unit variance that refers to mean centering and then divided by the standard deviation:

(3) $$x_{ik}^{\prime }=\frac{x_{ik}-{\bar{x}}_{k}}{s_{k}}$$

where $x_{ik}^{\prime }$ is the autoscaled response, $x_{ik}$ is the relative sensor response, ${\bar{x}}_{k}$ is the mean value of the normalized response for the specific sensor, NP represents the total number of samples, and $s_{k}$ is the standard deviation:

(4) $$s_{k}={\left[\frac{1}{\mathrm{NP}-1}\sum_{i=1}^{\mathrm{NP}}(x_{ik}-{\bar{x}}_{k}{)}^{2}\right]}^{1/2}$$

Autoscaling removes any inadvertent weighting that arises due to arbitrary units. After autoscaling, the value distribution of each sensor across the entire database was set to a mean value of zero and unit standard deviation [21]. 

### 2.5. Statistics

Using the pathological report as the reference standard, we calculated the accuracy of the breath test. Data collected in 2016 and 2017 were used to generate a prediction model, which was then randomly split into a training set (80%) for model derivation and a test set (20%) for internal validation. The subjects enrolled in 2018 were used as an independent dataset for external validation.

We applied both linear discriminant analysis (LDA) and non-linear support vector machine (SVM) learning techniques to build the prediction model. We assessed the diagnostic accuracy of the model by determining its sensitivity, specificity, false-positive rate, false-negative rate, accuracy, and area under the curve (AUC) with a corresponding 95% confidence interval (CI) of the receiver operating characteristic (ROC). All statistical analyses were conducted using MASS [24], the kernlab [25] package in R-3.4.4 software (R Foundation for Statistical Computing, Vienna, Austria) and IBM SPSS Statistics (version 20, IBM Corp., Armonk, NY, USA).

### 2.6. Sample Size Estimation

We used the following equation to calculate the required sample size [26]:

(5) $$\mathrm{SE}=\sqrt{\frac{C\left(100-C\right)}{n}}$$

where SE is the standard error, *C* is the percentage of patients classified correctly, and *n* is the estimated sample size. An SE of 3 was used to limit the standard error to no more than 3%, and the acceptable accuracy (*C*) was 80 based on our aim. The total sample size required for the training set was at least 178 patients.

## 3. Results

**Figure 2:**
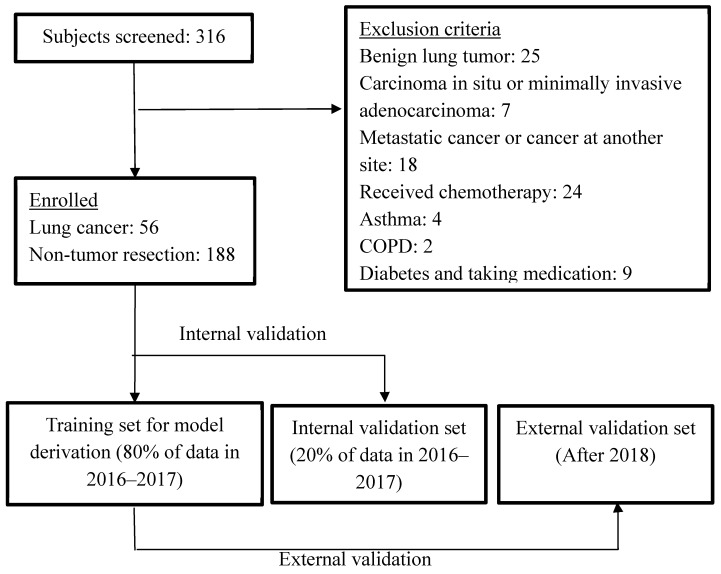
Flow diagram depicting the inclusion and exclusion of the study subjects. We employed an independent external validation set and conducted a repeated double cross-validation. The repeated double cross-validation used two nested loops. The inner loop used the study subjects enrolled between 2016 and 2017 as a calibration set for model selection and parameter optimization and were divided into a training set (80%) and an internal validation set (20%). The outer loop used the prediction model established from the calibration set to externally validate the study subjects enrolled in 2018.

**Figure 3:**
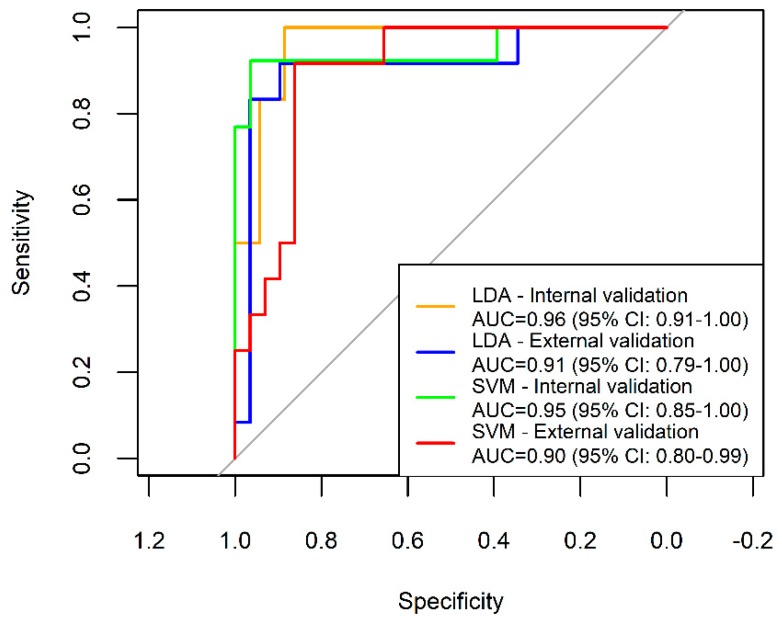
Receiver operating characteristic curves for lung cancers in the internal and external validation sets determined by LDA and SVM. The internal validation shows high accuracy by both linear and non-linear methods. The accuracy slightly decreases in the external validation.

We screened 265 subjects between 2016 and 2017. Based on the inclusion criteria, a total of 203 subjects were enrolled for the model derivation and internal validation. For the independent external validation set, we recruited 51 subjects in 2018. After excluding 10 subjects who had a benign lung tumour, metastatic lung cancer, carcinoma at another site, carcinoma in situ, or received chemotherapy, 41 subjects were used for the external validation, including 12 subjects with lung cancer and 29 controls (Figure 2). Table 1 shows the demographic characteristics of all the study subjects and the staging and histological types of the lung cancer patients. Most of the lung cancers were early-stage adenocarcinoma. The principal component analysis shows the discrimination between cases of lung cancer and controls (Supplementary Material, Figure S5). In the internal validation, the overall accuracies of both the LDA and SVM models were greater than 90%. In the external validation, the sensitivity, specificity, and overall accuracy of the SVM model were all greater than 80% (Table 2). The AUCs of the internal and external validation using LDA and SVM were all greater than 0.9 (Figure 3).

## 4. Discussion

This study showed that with the use of a highly sensitive (at the ppb level) chemical sensor and an advanced data analysis technique, the E-nose can be used to diagnose early-stage lung cancer with good accuracy.

We have carefully evaluated the validity of the breath test. Conductive polymers, quartz crystal microbalances, and metal oxide sensors are commonly used for the detection of lung cancer. In this study, we selected a conductive polymer sensor. This type of sensor is suitable to detect lipid peroxidation related VOCs, such as ethanol or isopropanol [12]. Instead of types of sensors, we found that the study design and selection of controls might have a greater influence on the detection accuracy than the type of sensor (Supplementary Material, Table S1). When the controls had other comorbidities, the diagnostic performance of the test was lower than that achieved when a healthy population was used as the control. Control heterogeneity, sometimes called “spectrum bias”, is usually observed in diagnostic tests using a case-control study design and may lead to an overoptimized accuracy [27]. In the E-nose development stage, studies must typically utilize a reliable test, such as LDCT or pathological reports, as a gold standard to test the performance of a new test. Therefore, most studies are conducted with a hospital-based case-control study design, and the accuracies obtained might thus not be directly applicable to communities wherein disease prevalence varies [28]. Validation is an essential step in the development of the prediction model. To assess the prediction accuracy, we have followed a strict repeated double cross-validation that uses two nested loops suggested by Marco [29]. The inner loop used the calibration set for model selection and parameter optimization using internal validation procedures, which is, dividing calibration set into a training set and an internal validation set. The outer loop was the division between the calibration set and an independent external validation set to estimate the prediction performance [29]. An external validation in an independent dataset from the targeted population that the screening method wants to apply is then warranted to ensure the repeatability of the test. In this study, we externally validated the breath test in our targeted population, and the test is then suitable for subjects from the same hospital. However, to apply the test to another population, a validation test is still necessary. 

We have paid attention to potential influence to sensors and evaluated sensors’ reliability. This study used a conductive polymer sensor, a type of chemiresistive sensor, which is sensitive to temperature, humidity [30], and baseline drift [12]. In this study, we operated the E-nose in the same room, which was maintained at a stable temperature between 20 and 25 °C and an environmental humidity between 50 and 65%. We also adopted a heat-moisture exchanger to the breath sampling device. In our pilot study, we measured the humidity of the sampled air before and after passage through the heat-moisture exchanger with a humidity meter (Rotronic HygroPlam, Bassersdorf, Switzerland, Supplementary Material, Figure S3). The mean relative humidity (R.H.) was 22.3% at the temperature of 24 °C. To prevent a sensor drift influence, we visually examined all 32 raw sensor responses of the 10 measurements. The 1st measurement was usually affected by the residual air in the connecting routes, and these data were therefore deleted. Upon visual examination of all the data, we deleted data that had significant drift. We also evaluated the repeatability of the sensor responses by calculating the intraclass correlation coefficients (ICC) between each measurement and the 2nd measurement with a 2-way fixed model (ICC 3, k model) [31]. The ICC values of the 32 sensors were all greater than 0.99, and the coefficients of variation (CVs) were within 0.08–0.32%, indicating excellent reliability (Supplementary Material, Table S2) [32].

All procedures in this study were standardized to prevent any factors that could influence the VOC concentration. To prevent contamination from the dead space in the respiratory or digestive tracts [33], we collected alveolar air from the endotracheal tube. All subjects were required to refrain from eating or smoking for 12 h before sampling. To prevent the influence from flow rate [18], we used a fixed flow rate to obtain a steady concentration of VOCs. To prevent the influence from contaminated sampling bag, we followed a standardized cleaning protocol according to recommendations from the European Respiratory Society [34]. Each bag was flushed with nitrogen five times and then heated to 45 °C for approximately 12 h; all the procedures were then repeated overnight, which has been shown to provide good recoveries [35]. Because the breath air samples were collected from anaesthetized subjects, they might have been influenced by anaesthesia drugs. However, all subjects were administered the same anaesthetic air (1–2% sevoflurane), and the anaesthetic dose was adjusted for each subject’s body weight (2 mg/kg for propofol, 2 μg/kg for fentanyl). We conservatively think the use of anesthetics might not confound our results.

Some studies support that volatile metabolites are generated from tumour tissue [36,37], while others suggest that VOCs are released from the systemic circulation and be released to the alveolar air by the gas exchange at the blood–gas interface in the lungs [38,39]. To determine the origin of volatile metabolites, we separately analysed VOCs from healthy and diseased lungs in the same patient among 24 subjects. After excluding two subjects with benign tumours, three subjects with metastatic lung cancer and two subjects in which both lungs were affected, 18 subjects were used for the analysis. However, the VOCs from healthy and diseased lungs were not discriminated well (Supplementary Material, Figure S4). These results are consistent with those of Capuano et al. [39], indicating that volatile biomarkers might be produced by not only tumour tissue but also by an epi-phenomenon that accompanies lung cancer development, probably due to the chronic load and burden of VOCs in overall lung tissue [40].

Though GC-MS can be used to precisely identify the chemical components, which is necessary to discover pathophysiological mechanisms, the E-nose can advantageously be used in clinical applications because it is simple to use, provides real-time analysis, and is hand-held in size. Moreover, the methods for analysing full-scan GC-MS signals have not been well established, and exhaled breath VOC libraries are currently being built but are not yet complete [41]. Untargeted analysis methods without standards often identify VOCs that are not replicable in different studies [42], and more studies are thus needed to explore actual biomarkers in the breath of lung cancer patients.

Use of sensors in the diagnosis of diseases is an emerging technology. The development of sensors is not limited to the breath test but also ingestible sensors [43]. A good collaboration between sensor engineers, medical doctors, and statisticians is important to accelerate the development of sensor technology in clinical use.

## 5. Conclusions

In this study, we used chemical sensors and a machine learning technique to develop a breath test for lung cancer. After all the procedure were standardized, the breath test developed herein had a high accuracy.

## Figures and Tables

**Table 1:** Demographic characteristics of the study subjects.

Characteristics	Lung Cancer Cases (*n* = 56)	Non-Tumour Controls (*n* = 188)
Age (year), mean (SD)	65.3 (8.8)	53.5 (16.1)
Male, no. (%)	12 (21.4)	106 (56.4)
Cigarette smoking		
Pack-years, mean (SD)	21.0 (10.7)	20.6(18.3)
Smoking status		
Current smokers, no. (%)	2 (3.6)	25 (13.3)
Former smokers, no. (%)	8 (14.3)	11 (5.9)
Never smoked, no. (%) ^a^	44 (78.6)	150 (79.8)
Second-hand smokers (%)	2 (3.6)	2 (1.1)
Tumour histological type		
Squamous cell carcinoma, no. (%)	1 (1.8%)	
Adenocarcinoma, no. (%)	52 (92.9%)	
Small cell lung cancer, no. (%)	1 (1.8%)	
Other carcinomas, no. (%)	2 (3.6%)	
Clinical stage		
I	37 (66.1%)	
II	7 (12.5%)	
III	11 (19.6%)	
IV	1 (1.8%)	

^a^ “Never smoked” means having smoked fewer than 20 packs of cigarettes in a lifetime or less than one cigarette per day for one year.

**Table 2:** Diagnostic accuracy of the E-nose.

Model	Sensitivity	Specificity	PPV	NPV	FP	FN	Accuracy
LDA internal validation	100.0%	88.6%	60.0%	100.0%	12.4%	0.0%	90.2%
LDA external validation	75.0%	96.6%	90.0%	90.3%	3.4%	25.0%	85.4%
SVM internal validation	92.3%	92.9%	85.7%	96.3%	7.1%	7.7%	92.7%
SVM external validation	83.3%	86.2%	71.4%	92.6%	13.8%	16.7%	85.4%

LDA, linear discriminant analysis; SVM, support vector machine; PPV, positive prediction rate; NPV, negative prediction value; FP, false-positive; FN, false-negative.

## Supplementary Materials

The following are available online at http://www.mdpi.com/1424-8220/18/9/2845/s1, Figure S1: Experimental setup for the analysis of alveolar air consisting of a (1) E-nose, (2) computer, (3) three-way valve and (4) Tedlar bag, Figure S2: Desired waveforms for Cyranose320, Figure S3: Instruments used to assess the humidity in the breath (Rotronic HygroPlam, Bassersdorf, Switzerland), Figure S4: Receiver operating characteristic curves for the discriminant from healthy and diseased lungs as determined by LDA and SVM, Figure S5: The principal component analysis shows the discrimination between cases of lung cancer and controls, Table S1: Comparison of diagnostic tests for lung cancer using gas sensor arrays, Table S2: Intraclass correlation coefficients (ICC) between each measurement (3rd–10th) and the second measurement (2nd) (*n* = 316) using the ICC (3, k) model. S1–32: sensors 1–32.
