# Supplementary material for: A Study of Diagnostic Accuracy Using a Chemical Sensor Array and a Machine Learning Technique to Detect Lung Cancer
Source: Sensors (Basel). 2018 Aug 28;18(9):2845. doi: 10.3390/s18092845 (PMC6164114; doi:10.3390/s18092845)
Supplement: Supplementary file 1 [file sensors-18-02845-s001.pdf]

A Study of Diagnostic Accuracy Using a Chemical Sensor Array and a Machine Learning Technique to Detect Lung Cancer

Chi-Hsiang Huang [1,2], Chian Zeng [3], Yi-Chia Wang [1,2], Hsin-Yi Peng [3], Chia-Sheng Lin [4], Che-Jui Chang [3,5] and Hsiao-Yu Yang [3,6*]

[1] Department of Anesthesiology, National Taiwan University College of Medicine, Taipei 10051, Taiwan; chhuang@ntu.edu.tw (C.-H.H.); yichiawang@ntu.edu.tw (Y.-C.W.)
[2] Department of Anesthesiology, National Taiwan University Hospital, Taipei 10048, Taiwan
[3] Institute of Occupational Medicine and Industrial Hygiene, National Taiwan University College of Public Health, Taipei 10055, Taiwan; michellzeng0912@gmail.com (C.Z.); yope159@gmail.com (H.-Y.P.); L1204366@gmail.com (C.-J.C.)
[4] Department of Clinical Laboratory Sciences and Medical Biotechnology, National Taiwan University, Taipei 10051, Taiwan; b04404046@ntu.edu.tw (C.,-S. L.)
[5] Department of Family Medicine, National Taiwan University Hospital, Taipei 10048, Taiwan
[6] Department of Public Health, National Taiwan University College of Public Health, Taipei 10055, Taiwan
* Correspondence: hyang@ntu.edu.tw (H.,-Y.Y.); Tel.: +886-2-3366-8102

Table S1. Comparison of diagnostic tests for lung cancer using gas sensor arrays.

Author	Year	Cancer subjects	Control subjects	Breath sampling	Sensor	Temperature/humidity	Use of validation	Accuracy
Di Natale et al. [1]	2003	35	18	Mix air	Quartz crystal microbalance	Unknown	Internal	Sensitivity: 100%<br>Specificity: 94%
Machado et al. [2]	2005	14	62 (other disease)	Mix air	Conductive polymer	Unknown	External	Sensitivity: 71.4%<br>Specificity: 91.9%
Mazzone et al. [3]	2007	49	94 (other disease)	Mix air	Chemically sensitive spots	Unknown	Internal	Sensitivity: 73.3%<br>Specificity: 72.4%
Dragonieri et al. [4]	2009	10	10	Mix air	Conductive polymer	Unknown	Internal	Accuracy: 90%
D'Amico et al. [5]	2010	28	36	Alveolar air	Quartz crystal microbalance	Unknown	Internal	Sensitivity: 85%<br>Specificity: 100%
Santonico et al. [6]	2012	20	10	Alveolar air (bag breath sampling)	Quartz crystal microbalance	Unknown	Internal validation	Sensitivity: 85%<br>Specificity: 85%
				Alveolar air (endoscopic breath sampling)			Internal validation	Sensitivity: 97.5%<br>Specificity:75%
Hubers et al. [7]	2014	18	8	Mix air	Conductive polymer	Unknown	Internal	Sensitivity: 94%<br>Specificity: 13%
Rocco et al. [8]	2016	23	77	Mix air	Acoustic-mass	20~22°C	Internal	Sensitivity: 86%<br>Specificity: 95%

Gasparri et al. [9]	2016	70	76	Alveolar air	Quartz crystal microbalance	Unknown	Internal	Sensitivity: 81%<br>Specificity: 91%
Tirzīte et al. [10]	2017	165	79	Mix air	Conductive polymer	Unknown	Internal	Sensitivity: 97.8%<br>Specificity: 68.8%
Van de Goor et al. [11]	2018	52 (training set)	93 (training set)	Mix air	Metal oxide	Unknown	Internal	Sensitivity: 83%<br>Specificity: 84%
		8 (validation set)	14 (validation set)				External	Sensitivity: 88%<br>Specificity: 86%
Chang et al. [12]	2018	37	48	Alveolar air	Metal oxide	Unknown	Internal	Sensitivity: 79%<br>Specificity: 72%

Table S2. Intraclass correlation coefficients (ICC) between each measurement (3rd–10th) and the second measurement (2nd) (n =316) using the ICC (3, k) model. S1–32: sensors 1–32.

Sensor	Measurement							
	3rd	4th	5th	6th	7th	8th	9th	10th
S1	1	1	1	0.999	0.999	0.999	0.999	0.999
S2	1	1	1	1	1	1	0.999	0.999
S3	1	1	1	1	1	0.999	0.999	0.999
S4	1	0.999	0.999	0.999	0.999	0.998	0.998	0.998
S5	1	0.999	0.998	0.997	0.996	0.995	0.994	0.993
S6	0.998	0.994	0.993	0.99	0.988	0.987	0.987	0.986
S7	1	0.999	0.999	0.999	0.998	0.998	0.998	0.997
S8	0.996	0.995	0.994	0.994	0.994	0.993	0.993	0.993
S9	1	1	1	0.999	0.999	0.999	0.999	0.999
S10	0.999	0.998	0.997	0.996	0.994	0.994	0.993	0.992
S11	0.999	0.999	0.998	0.997	0.997	0.996	0.995	0.995
S12	1	0.999	0.999	0.998	0.997	0.997	0.997	0.996
S13	1	0.999	0.999	0.999	0.998	0.998	0.998	0.997
S14	1	1	1	1	0.999	0.999	0.999	0.999
S15	0.999	0.999	0.998	0.997	0.996	0.995	0.995	0.995
S16	1	0.999	0.999	0.998	0.998	0.997	0.997	0.996
S17	1	0.999	0.999	0.999	0.998	0.998	0.998	0.997
S18	0.999	0.996	0.996	0.994	0.993	0.993	0.993	0.991
S19	1	0.999	0.999	0.999	0.998	0.998	0.997	0.997
S20	1	1	0.999	0.999	0.999	0.999	0.998	0.998
S21	1	0.999	0.999	0.998	0.998	0.998	0.997	0.997
S22	0.999	0.999	0.998	0.997	0.995	0.994	0.994	0.992
S23	1	0.999	0.998	0.998	0.997	0.996	0.996	0.995
S24	0.999	0.999	0.998	0.998	0.997	0.997	0.997	0.996
S25	1	0.999	0.999	0.998	0.998	0.998	0.997	0.997
S26	1	1	0.999	0.999	0.999	0.998	0.998	0.998
S27	1	1	0.999	0.999	0.999	0.999	0.999	0.998
S28	1	1	1	0.999	0.999	0.999	0.999	0.998
S29	1	1	1	0.999	0.999	0.999	0.999	0.999
S30	1	0.999	0.999	0.998	0.998	0.998	0.997	0.997
S31	0.999	0.998	0.997	0.996	0.994	0.992	0.991	0.989
S32	1	1	0.999	0.999	0.999	0.999	0.998	0.998
Mean	1.000	0.999	0.998	0.998	0.997	0.997	0.997	0.996
± SEM	±0.001	±0.001	±0.002	±0.002	±0.003	±0.003	±0.003	±0.003
CV(%)	0.08%	0.14%	0.16%	0.21%	0.25%	0.28%	0.28%	0.32%

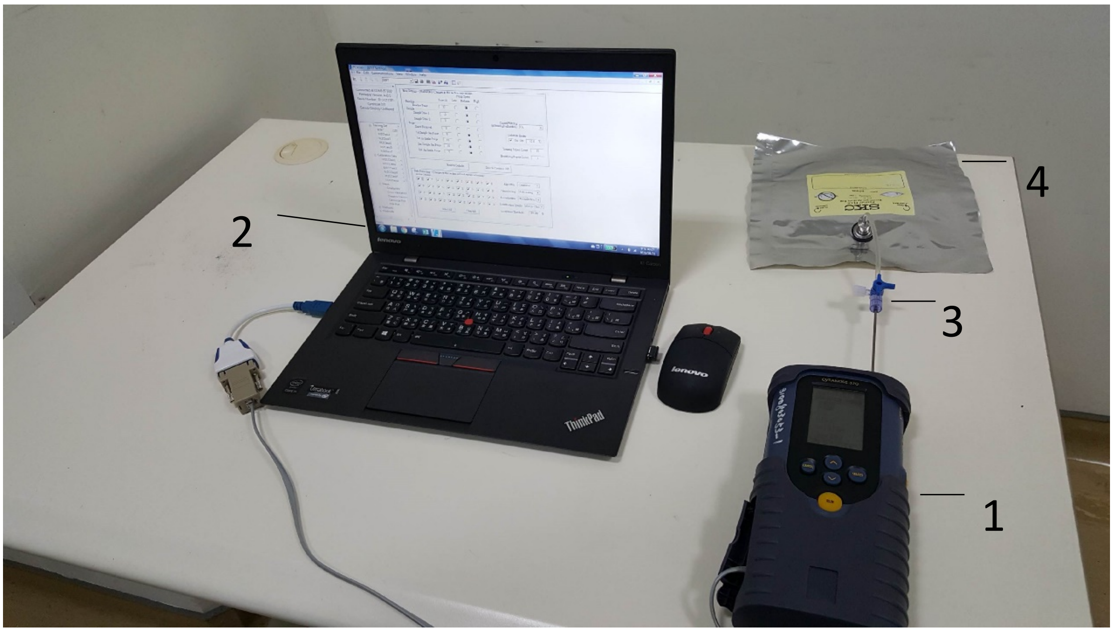

Figure S1. Experimental setup for the analysis of alveolar air consisting of a (1) E-nose, (2) computer, (3) three-way valve and (4) Tedlar bag.

Legend: The bags were connected with the necessary fixture, including an airtight PVC tube and a three-way valve for connection to the E-nose.

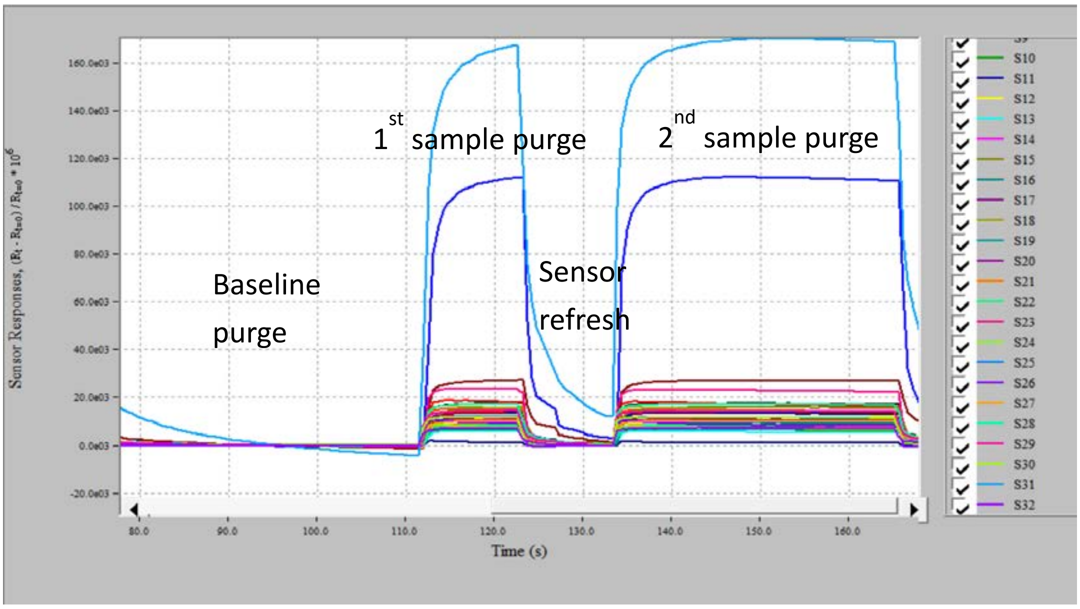

Figure S2. Desired waveforms for Cyranose320.

Legend: The setting comprised 10 seconds of a baseline purge and 40 seconds of a sample purge, which was sufficient for most sensors to reach the steady-state, followed by 10 seconds of a wash-out to return to the baseline.

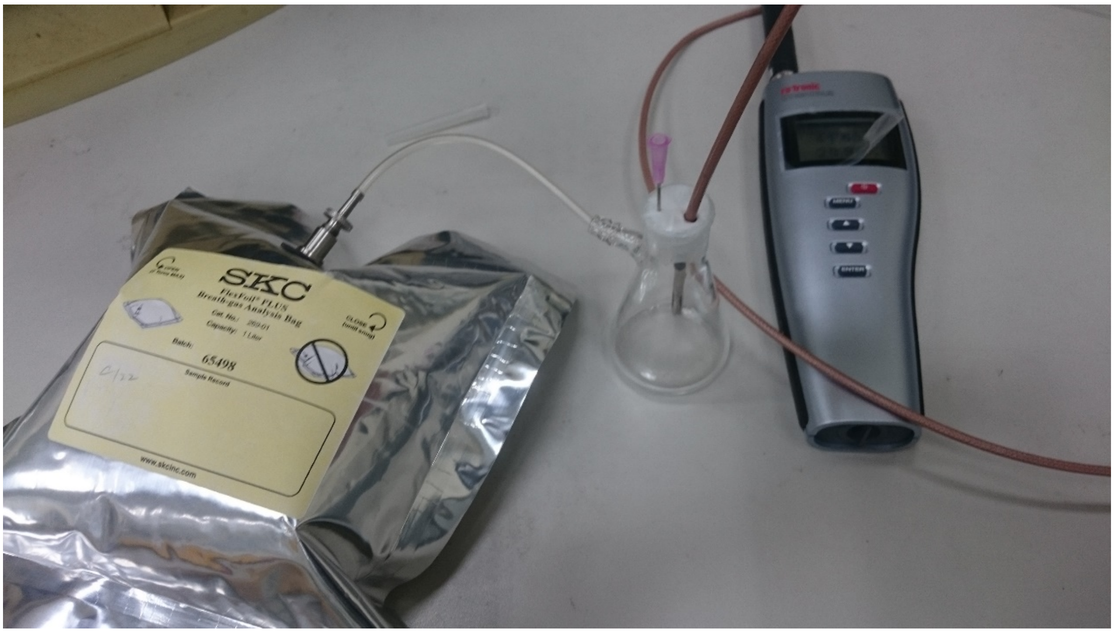

Figure S3. Instruments used to assess the humidity in the breath (Rotronic HygroPlam, Bassersdorf, Switzerland).

Legend: We used a humidity meter to measure the humidity in the Tedlar bag, and the mean humidity was 22.32% R. H. (R. H. was measured at 24°C).

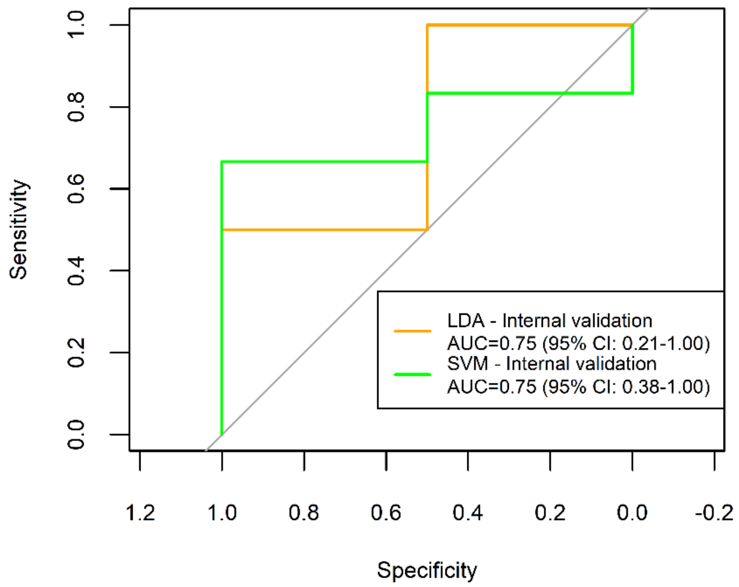

Figure S4. Receiver operating characteristic curves for the discriminant from healthy and diseased lungs as determined by LDA and SVM.

Legend: The VOCs collected from healthy and diseased lungs in the same subjects cannot be discriminated well by both linear and non-linear statistical methods.

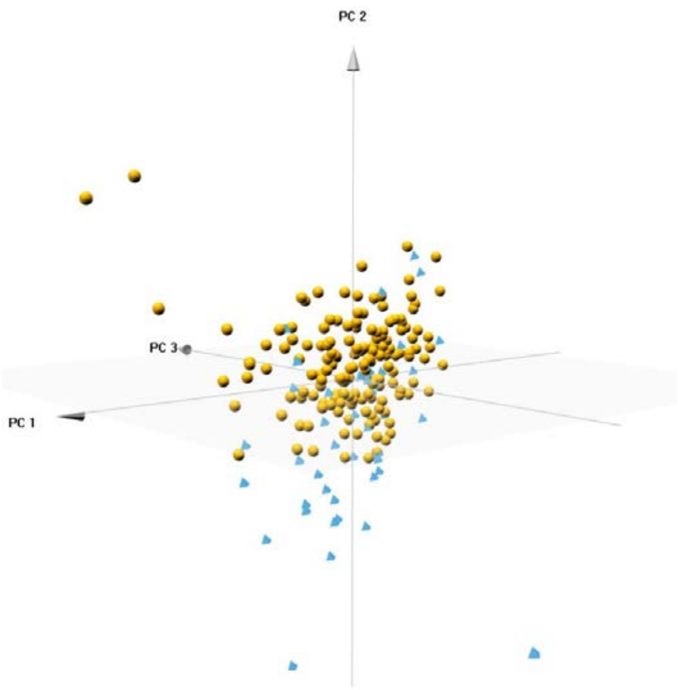

Figure S5. The principal component analysis shows the discrimination between cases of lung cancer and controls.

Legend: The yellow points in the right upper corner are controls, and the blue triangles in the lower portion are lung cancer cases. Using three principal components, cases and controls could be discriminated well.

References

1. Di Natale, C.; Macagnano, A.; Martinelli, E.; Paolesse, R.; D'Arcangelo, G.; Roscioni, C.; Finazzi-Agro, A.; D'Amico, A. Lung cancer identification by the analysis of breath by means of an array of non-selective gas sensors. *Biosens. Bioelectron.* **2003,** *18*, 1209–1218.
2. Machado, R.F.; Laskowski, D.; Deffenderfer, O.; Burch, T.; Zheng, S.; Mazzone, P.J.; Mekhail, T.; Jennings, C.; Stoller, J.K.; Pyle, J.; Duncan, J.; Dweik, R.A.; Erzurum, S.C. Detection of lung cancer by sensor array analyses of exhaled breath. *Am. J. Respir. Crit. Care. Med.* **2005,** *171*, 1286–1291.
3. Mazzone, P.J.; Hammel, J.; Dweik, R.; Na, J.; Czich, C.; Laskowski, D.; Mekhail, T., Diagnosis of lung cancer by the analysis of exhaled breath with a colorimetric sensor array. *Thorax* **2007,** *62*, 565–568.
4. Dragonieri, S.; Annema, J.T.; Schot, R.; van der Schee, M.P.; Spanevello, A.; Carratu, P.; Resta, O.; Rabe, K.F.; Sterk, P.J. An electronic nose in the discrimination of patients with non-small cell lung cancer and COPD. *Lung Cancer* **2009,** *64*, 166–170.
5. D'Amico, A.; Pennazza, G.; Santonico, M.; Martinelli, E.; Roscioni, C.; Galluccio, G.; Paolesse, R.; Di Natale, C. An investigation on electronic nose diagnosis of lung cancer. *Lung Cancer* **2010,** *68*, 170–176.
6. Santonico, M.; Lucantoni, G.; Pennazza, G.; Capuano, R.; Galluccio, G.; Roscioni, C.; La Delfa, G.; Consoli, D.; Martinelli, E.; Paolesse, R.; Di Natale, C.; D'Amico, A., In situ detection of lung cancer volatile fingerprints using bronchoscopic air-sampling. *Lung Cancer* **2012,** *77*, 46–50.
7. Hubers, A.J.; Brinkman, P.; Boksem, R.J.; Rhodius, R.J.; Witte, B.I.; Zwinderman, A.H.; Heideman, D.A.; Duin, S.; Koning, R.; Steenbergen, R.D.; Snijders, P.J.; Smit, E.F.; Sterk, P.J.; Thunnissen, E. Combined sputum hypermethylation and eNose analysis for lung cancer diagnosis. *J. Clin. Pathol.* **2014,** *67*, 707–711.
8. Rocco, R.; Incalzi, R.A.; Pennazza, G.; Santonico, M.; Pedone, C.; Bartoli, I.R.; Vernile, C.; Mangiameli, G.; La Rocca, A.; De Luca, G.; Rocco, G.; Crucitti, P. BIONOTE e-nose technology may reduce false positives in lung cancer screening programmesdagger. *Eur. J. Cardiothorac. Surg.* **2016,** *49*, 1112-1117.
9. Gasparri, R.; Santonico, M.; Valentini, C.; Sedda, G.; Borri, A.; Petrella, F.; Maisonneuve, P.; Pennazza, G.; D'Amico, A.; Di Natale, C.; Paolesse, R.; Spaggiari, L. Volatile signature for the early diagnosis of lung cancer. *J. Breath Res.* **2016,** *10*, 016007.
10. Tirzīte, M.; Bukovskis, M.; Strazda, G.; Jurka, N.; Taivans, I., Detection of lung cancer in exhaled breath with an electronic nose using support vector machine analysis. *J. Breath Res.* **2017,** *11*, 036009.
11. Van de Goor, R.; Van Hooren, M.; Dingemans, A.M.; Kremer, B.; Kross, K., Training and validating a portable electronic nose for lung cancer screening. *J. Thorac. Oncol.* **2018,** *13*, 676-681.
12. Chang, J.-E.; Lee, D.-S.; Ban, S.-W.; Oh, J.; Jung, M.Y.; Kim, S.-H.; Park, S.; Persaud, K.; Jheon, S., Analysis of volatile organic compounds in exhaled breath for lung cancer diagnosis using a sensor system. *Sens. and Actuators B: Chem.* **2018,** *255*, 800–807.

© 2018 by the authors. Submitted for possible open access publication under the terms and conditions of the Creative Commons Attribution (CC BY) license (http://creativecommons.org/licenses/by/4.0/).
